# Validity and Reproducibility of a Self-Administered Food Frequency Questionnaire for the Assessment of Sugar Intake in Middle-Aged Japanese Adults

**DOI:** 10.3390/nu11030554

**Published:** 2019-03-05

**Authors:** Rieko Kanehara, Atsushi Goto, Ayaka Kotemori, Nagisa Mori, Ari Nakamura, Norie Sawada, Junko Ishihara, Ribeka Takachi, Yukari Kawano, Motoki Iwasaki, Shoichiro Tsugane, for the JPHC FFQ Validation Study Group

**Affiliations:** 1 Epidemiology and Prevention Group, Centre for Public Health Sciences, National Cancer Centre, Tokyo 104-0045, Japan; rkanehar@ncc.go.jp (R.K.); asunami@ncc.go.jp (A.K.); nagmori@ncc.go.jp (N.M.); ar-nakamura@my-zaidan.or.jp (A.N.); nsawada@ncc.go.jp (N.S.); j-ishihara@azabu-u.ac.jp (J.I.); rtakachi@cc.nara-wu.ac.jp (R.T.); moiwasak@ncc.go.jp (M.I.); stsugane@ncc.go.jp (S.T.); 2 Department of Food and Nutritional Science, Graduate School of Agriculture, Tokyo University of Agriculture, Tokyo 156-8502, Japan; 3 Department of Food and Life Science, School of Life and Environmental Science, Azabu University, Kanagawa 252-5201, Japan; 4 Department of Food Science and Nutrition, Faculty of Human Life and Environment, Nara Women’s University, Nara 630-8506, Japan; 5 Department of Nutritional Science, Faculty of Applied Bioscience, Tokyo University of Agriculture, Tokyo 156-8502, Japan; y1kawano@nodai.ac.jp

**Keywords:** food frequency questionnaire, sugar intakes, dietary record, East Asians

## Abstract

We evaluated the validity and reproducibility of estimated sugar intakes using a food frequency questionnaire (FFQ) among middle-aged Japanese adults in the Japan Public Health Centre-Based Prospective (JPHC) study. In subsamples of the JPHC study (Cohorts I and II in multiple areas), we computed Spearman’s correlations of FFQ results with urine sugar concentrations and dietary records (DR) for validity; we evaluated correlations between two FFQs for reproducibility. During 1994–1998, participants (Cohort I: *n* = 27 [men], *n* = 45 [women]) provided two (spring and fall) 24-h urine samples and completed 7-consecutive-day DR per season (I: *n* = 99, *n* = 113; II: *n* = 168, *n* = 171) and two FFQs (147 food items) at yearly intervals (I: *n* = 101, *n* = 108; II: *n* = 143, *n* = 146). Sugar intakes from FFQ were correlated with urinary sugar (de-attenuated correlations: 0.40; 95%CI: 0.19, 0.58). After adjustment for sociodemographic and lifestyle variables, correlations between FFQ and DR for men and women were 0.57 (0.42, 0.69) and 0.41 (0.24, 0.55) (I) and 0.56 (0.44, 0.65) and 0.34 (0.20, 0.47) (II), respectively. Correlations between FFQs for men and women were 0.63 (0.49, 0.73) and 0.55 (0.41, 0.67) (I) and 0.66 (0.55, 0.74) and 0.63 (0.52, 0.72) (II). In conclusion, our study showed moderate FFQ validity and reproducibility for sugar intake evaluation.

## 1. Introduction

The prevalence of obesity and chronic diseases, such as diabetes, is rising steadily worldwide [1,2], leading to increased financial burden from medical expenses and the need to identify preventive measures urgently. The potential role of dietary sugar (especially free or added sugars) consumption in the development of these health conditions has drawn much attention. The World Health Organization (WHO) recommended in the guideline for sugar intake that the intake of free sugars (added or processed sugars, and sugars in honey, syrups, and fruits juices), should be less than 10% of the energy intake [3]. Previous studies, primarily among Westerners, examined associations between the consumption of sugars (mono- and di-saccharide; fructose, glucose, and sucrose) and chronic diseases or conditions, suggesting that the overconsumption of free sugars may lead to chronic diseases [4,5,6]. Among Japanese populations, however, few previous studies [7,8] have examined the associations between sugars and health conditions. Moreover, according to previous studies on estimations of sugar intakes [9,10], amount and source of dietary sugar consumption among Japanese populations may differ from that among European populations. Owing to these differences between Japanese and Europeans, health impacts of sugar intakes may also differ. Hence, the impact of sugar intakes on the health of the Japanese population merits further investigation.

In Japan, the Ministry of Education, Culture, Sports, Science, and Technology published standard tables of detailed food composition for carbohydrates in 2015 [11]. This further served as a motivation to quantify the dietary intake of sugars among Japanese populations. The food frequency questionnaire (FFQ) is widely used and is less burdensome as a dietary assessment method among study participants than other methods, such as the dietary record (DR). However, it is necessary to verify whether the health impact of nutrient intakes can be accurately estimated using the FFQ [12]. The Japan Public Health Centre-Based Prospective (JPHC) study [13] is a large-scale, nationwide, population-based cohort study with a follow-up period of over 20 years, since its establishment in 1990. In a subsample of the JPHC study, we examined the validity of sugar intakes estimated based on the FFQ, by comparing urinary sugar concentrations as an objective biomarker and DR results for 7 consecutive days per season (28- or 14-d). In addition, the reproducibility was compared using two FFQs completed at a yearly interval.

## 2. Materials and Methods

### 2.1. JPHC Validation Study and Participants

The Japan Public Health Centre-Based Prospective (JPHC) study is a prospective cohort study conducted on men and women aged 40 to 69 years. Cohorts I (since 1990) and II (since 1993) were living in five (Ninohe, Yokote, Saku, Ishikawa, and Katsushika) and six (Mito, Kashiwazaki, Chuo-higashi, Kamigoto, Miyako, and Suita) public health centre (PHC) areas, respectively. A 5-year follow-up study was conducted in 1995 (Cohort I) and 1998 (Cohort II) using the FFQ. The FFQ was developed based on weighed 3-d DR survey data from Cohort I participants. Validation studies, for the FFQ, and described previously [14,15], were carried out among a subsample of participants in the JPHC Study Cohorts I and II.

In brief, the Cohort I validation study was performed from February 1994 to February 1996 while Cohort II was performed from May 1996 to February 1998. Participants completed 28 d (14 d for Ishikawa PHC area) DR, they also completed the FFQ twice, while some in Cohort I also collected 24-h stored urine. The FFQ, completed by participants after 3 months of completing the DR, was used for the validation (FFQv). Participants also completed another FFQ (FFQr) at yearly intervals (9-month interval for Mito PHC area) that was used to determine reproducibility. Sample size calculations revealed that approximately 112 participants would be required to detect a CC of 0.25 with α = 0.05 and β = 0.20 separately for men and women, and Cohorts I and II. A total of 120 married couples in Cohort I and 196 married couples in Cohort II were recruited. The participants or their spouses who were out of the age range for the cohorts were excluded. Furthermore, data of the participants without a complete 28 d (14 d for Ishikawa PHC area) DR or FFQv were excluded from validation, while those without a complete FFQr were excluded from reproducibility. Thus, data from a total of 215 participants (102 men and 113 women) from Cohort I and 350 participants (174 men and 176 women) from Cohort II were included for the validation between DR and FFQv. For the calculation of partial correlation coefficients, we further excluded those who had missing data for occupation, smoking status or alcohol intake, leaving a total of 212 participants (99 men and 113 women) from Cohort I and 339 participants (168 men and 171 women) from Cohort II. Furthermore, 72 participants (27 men and 45 women) were included for the validation between the biomarker and FFQv or DR. From Cohort I, 209 participants (101 men and 108 women) and Cohort II, 289 participants (143 men and 146 women) were included for the reproducibility between FFQv and FFQr [14,15].

All participants gave their oral or written informed consent for participation in the JPHC validation study. The protocol for the current study, including data analysis and the measurement of urinary sugar concentrations, was conducted according to the guidelines laid down in the Declaration of Helsinki and approved by the human ethics review committee of the National Cancer Centre of Japan (No. 2016-428).

### 2.2. Food Frequency Questionnaire in the 5-Year Follow-Up Survey

The FFQ (which included 147 food items) required information about the usual food consumption during the previous year. Basically, questions about portion size (<0.5 (small)/one (medium)/>1.5 (large) times the reference amount) and frequency (almost never, one to three times per month, one to two times per week, three to four times per week, five to six times per week, once per day, two to three times per day, four to six times per day, and seven or more times per day) were asked. Further questions about consumptions of rice (bowl size/number of bowls per day/consumptions of vitamin reinforced rice and millet), miso soup (number of days eaten per week or month/number of bowls per day/taste intensity), alcohol (number of days drank per week or month/amount per day and types of liquor), supplements (number of tablets per day or week/period), were asked. Additionally, the added sugar and milk for coffee and tea, the usual cooking method, and the amount of noodles soup consumed were also enquired.

### 2.3. Biomarker for Sugar Intakes

Of 215 participants who completed the DR and the FFQv in Cohort I (the cohort used in developing the FFQ) [16], 72 collected their urine for 24 h. The urine collections were performed for two days (on any day during the 7-d DR period, once in spring and fall). After recording the total volume of the urine collected in a portable device (Urine Mate P, Sumitomo Bakelite, Tokyo, Japan), the urine samples were frozen and stored at −80 °C [14]. Cohort II participants were not asked to provide their urine samples.

Concentrations of sucrose and fructose in the urine (μg/mL) were measured with a kit (F-kit Sucrose/d-glucose/d-Fructose; Roche/R-Biopharm AG, Darmstadt, Germany) and NanoDrop ND-1000 Spectrophotometer (NanoDrop Technologies, Wilmington, DE, USA). A total of 144 samples (72 participants per season for 2 seasons) were analysed. For quality control, four samples were measured twice, and the quality of the method was assessed. Intra-assay coefficients of variation (CVs) were 4.2% or lower. Other samples were measured once.

### 2.4. Dietary Records

A total of 565 participants in Cohorts I and II completed the 7 consecutive DR days over each of the four seasons (two seasons [winter and summer], were used for the Ishikawa PHC area because of the subtropical climate during which seasonal variations were likely to be brief). The participants were instructed by the research dietitians to record all foods and beverages prepared and consumed; using a specially developed booklet, they were asked to describe, in as much detail as possible, the methods and recipes used in the preparation. The dietitians checked the records during the survey and reviewed them in a standardized way. Details have been reported elsewhere [14,15].

### 2.5. Food Composition Table of Carbohydrates, and Nutritional Calculation for FFQ and DR

The 2015 standard tables of food composition in Japan for available carbohydrates include monosaccharides (glucose, fructose, and galactose), disaccharides (sucrose, maltose, lactose, and trehalose), and polysaccharide (starch), and these can be digested and absorbed in the human body [11]. Carbohydrates cover 854 of all 2191 food items in the 2015 standard tables of food composition in Japan [17]. Intakes of glucose, fructose, galactose, sucrose, maltose, lactose, total sugars (sum of these six mono- or disaccharides), and starch from the FFQ and the DR were calculated using the standard tables of food composition for the available carbohydrates [11].

In the FFQ, 75 of 147 food items were covered by the table while 72 were not. Eighteen food items (Table S1) were substituted for by the following methods [18] using different parts of the same species, similar species, or same species with different cooking or purification methods. Among the 54 remaining food items, 48 with <1 g of carbohydrates available per portion size were regarded as containing no carbohydrate. Finally, the remaining six foods items (Table S1) were prepared by dietitians (A.K. and R.K.) using the recipes that were based on the ingredient blending ratio from food manufacturers, cookbooks, and the component values of proteins, lipids, and carbohydrates listed in the appendix in the 2015 Japan standard tables of food composition.

For the FFQ, we also included sugars added to foods during cooking in the calculations by preparing a recipe of the main menus: sugar intakes from table sugar, miso, soy sauce, cooking sake, and sweet cooking rice wine (mirin). First, the main DR menu (e.g., boiled chub mackerel) for each food group (meat, fish, vegetable) and each cooking method (raw, simmered, grilled, fried, stir-fried, others) were selected to cover > 80% of the DR frequent food items. Secondly, selected menu recipes were prepared by dietitians as described above, and sugar intakes were calculated for each of the menu. Thirdly, we calculated the weighted average values of sugar intakes for each of the classifications based on the frequency of occurrence of the menus in the DR because there were multiple menus in the same classification of dishes. For meat (beef, pork, and chicken), the values of sugar intake were calculated using food menus. For fish and vegetable, the values of sugar intake were calculated based on the cooking method (raw, simmered, grilled, fried, stir-fried, and others).

In the DR, a total of 1241 food items were recorded. Of these, 743 were not included in the 2015 standard tables of food compositions for the available carbohydrates. Among food items not included in the table, we substituted 141 foods with different parts of the same species, similar species, same species with different cooking or purification methods (119 food items), or recipes prepared by dietitians (22 food items). The 141 foods included cereals, sugars and sweeteners, pulses, nuts and seeds, vegetables, fruits, milk and milk products, confectionaries, beverages, seasonings and spices, and prepared foods. The remaining food items (602: some vegetables and fruits, mushrooms, algae, fish, meat, eggs, oils and fat, beverages, and seasonings and spices) were not substituted by any other foods. Only twenty-six out of 602 food items contained more than 5 g available carbohydrate, and frequencies of consumption for these foods were extremely low. Therefore, they were considered to have little contribution to the total sugar consumption.

Intakes of energy, protein, fat, and carbohydrate from the FFQ and the DR were calculated using the 2015 Japan standard tables of food composition [17] for reference.

### 2.6. Statistical Analysis

Major food groups contributing to sugar intakes, by gender, were identified by sugar intakes from the DR. The mean intake and standard deviation (mean ± SD) of glucose, fructose, galactose, sucrose, maltose, lactose, total sugars (sum of these six mono or disaccharides), starch, energy, protein, fat, and carbohydrate from the FFQ and the DR were calculated by gender and by cohort groups. Differences were calculated using the following formula: intakes according to the FFQ—intakes according to the DR. Mean and 95% confidence interval (95%CI) of the differences were calculated. Spearman’s rank CCs and Pearson CCs between the FFQv and DR (for validity), and between the FFQv and FFQr (for reproducibility), were calculated for crude and energy-adjusted values of sugar and macronutrient intakes. Correlation coefficients calculated with 95%CI using Fisher’s z-transformation.

Energy-adjusted values were estimated using the residual and nutritional density methods. Nutritional density (% energy) was calculated with the following formula: energy intake from sugars/total energy intake × 100. The metabolized energy conversion factor (General Atwater factor) for monosaccharides is 3.75, and the conversion factor from disaccharides to monosaccharides is 1.05 [19]. Moreover, for validity, partial CCs adjusted for age, areas, occupations (primary industry, professionals and office workers, self-employed and others, unemployed), body mass index (BMI), total energy intake, smoking status (never, past, current), and alcohol (nondrinker, ≤4 days per week, ≥5 days per week) were calculated.

Urinary sugars have been suggested as a useful biomarker to estimate the total sugar intakes, independent of measurement errors from self-reported measures [20]. Spearman’s rank CCs and Pearson CCs were calculated to compare between DR or FFQv and the sum of the urinary concentration of sucrose and fructose (urinary sugars). To compare between FFQv and urinary sugars, we used total sugar intakes from the FFQv and the mean urinary concentrations of sugars collected in the spring and fall. For the DR, we compared sugar intakes from the mean 14-d DR in spring and fall with urinary concentrations of sugars, and also compared the mean 7-d DR with urinary sugars separately for the spring and fall. CCs were reported for crude values, energy-adjusted values (for the DR and the FFQv, % energy), and creatinine-adjusted values (for urinary sugars, divided by urinary creatinine concentration (mg/dL)). Furthermore, scatter plots between urinary sugars (creatinine-adjusted) and sugar intakes (%energy) from the FFQ and mean 14-d DR are shown.

Additionally, to correct for within-individual random error, energy-adjusted (% energy) or creatinine-adjusted Spearman’s CCs (comparing the FFQv vs. DR; and the urinary sugars vs. the FFQv and DR) were de-attenuated based on the method in SAS macro (“rankcorr_mmer.sas”) provided by Dr. Bernard Rosner [12,21,22] using probit transformation and multiplying each with the adjustment factor. The adjustment factors were calculated by using the following formula for FFQv vs. DR and FFQv vs. urinary sugars: $\sqrt{1+\frac{\lambda }{k}}$, where *k* is the average DR days for FFQv vs. DR; or the frequency of the urine collection for FFQv vs. urinary sugars; and *λ* is the ratio of within- to between-subject variance within the 14- or 28-day DR or urinary sugars collected twice, using the random-effects model [12]. For urinary sugars vs. the DR, the adjustment factor was taken into account for the within-individual random errors in measurement of both the DR and urinary sugars. The formula of the adjustment factor was the following: $\sqrt{1+\frac{\lambda_{dr}}{k_{dr}}}\times \sqrt{1+\frac{\lambda_{urine}}{k_{urine}}}$, where *k_dr_* is the average DR days and *k_urine_* is the frequency of the urine collection; and *λ_dr_* is the ratio of within- to between-subject variance within the 14- or 28-day DR and *λ_urine_* is for urinary sugars [12].

To evaluate intra-subject variations for urinary sugars, the ratio of within- to between-subject variance (σ^2^_ws_/σ^2^_bs_) and intra-class CCs [ICC; σ^2^_bs_/(σ^2^_bs_ + σ^2^_ws_)] of urinary sugars collected in the spring and fall were calculated, using the random-effects model [12]. ICCs of the two FFQs and 28- or 14-d DR were also calculated.

In addition, the proportion of participants, who were classified into the same, adjacent, and extreme categories using the cross classification by quintile [23] for energy-adjusted (% energy) total sugar intakes or creatinine-adjusted urinary sugar, was calculated. The adjacent categories included the proportion of participants who were not in the same category by quintile between the two measurement methods (the FFQv vs. DR or the FFQv vs. urinary sugar), but only in the +1 or −1 difference categories. The extreme categories included the proportion of participants who were misclassified into the opposite side class (for example, the class for the FFQv was the highest, but the DR’s was the lowest).

Agreement between total sugar intakes from the FFQv and DR were examined using Bland-Altman analysis. We plotted the mean total sugar intakes from the FFQv and DR on the *x*-axis, and the difference between them (FFQv and DR) on the *y*-axis using energy-adjusted (% energy) and log-transformed values. Mean difference ± 1.96 × SD was calculated as the limit of agreement [24,25,26].

For parametric methods such as Pearson CCs and the Bland-Altman analysis, all nutrient intake values were log-transformed to fulfill the assumption of normality. Statistical significance was set at a *p* value of <0.05. All statistical analyses were implemented in SAS version 9.3.

## 3. Results

For sugar intakes, contribution proportions by food groups were calculated from the DR (men: *n* = 276, women: *n* = 289). Contribution proportions of fruits, mostly from apples, citrus, bananas, and Japanese persimmons, were the highest for total sugars in both men and women. For women, the proportion of confectioneries in total sugars was higher than that for men (Table 1). In detail, contribution proportions by foods for each of the mono- and di-saccharides and starch are shown in Table S2. The mean (SD) of % energy for total and free sugars was 9.5% (3.3%) and 3.9% (2.3%) for men (*n* = 276), 13.6% (3.2%) and 5.9% (2.3%) for women (*n* = 289). For free sugars, the number of participants who consumed more than 5% was 68 (24.6%) in men and 186 (64.4%) in women. Furthermore, the number of participants who consumed more than 10% was 6 (2.2%) in men and 16 (5.5%) in women.

For validation, participants’ characteristics were described in previous studies [14,15]. In short, the mean (SD) age and BMI were 55.6 (5.2) years and 24.3 (3.0) kg/m^2^ for men in Cohort I (*n* = 102); 53.3 (5.3) years and 23.9 (3.1) kg/m^2^ for women in Cohort I (*n* = 113); 58.9 (7.6) years and 23.7 (2.6) kg/m^2^ for men in Cohort II (*n* = 174); and 55.9 (7.1) years and 23.7 (3.2) kg/m^2^ for women in Cohort II (*n* = 176). The percentages of participants who had history of diabetes, hypertension, dyslipidaemia, and obesity (BMI ≥ 25 kg/m^2^) were 7.8%, 18.6%, 5.9%, and 42.2% for men in Cohort I; 3.5%, 22.1%, 8.0%, and 31.0% for women in Cohort I; 8.1%, 20.1%, 4.6%, and 28.2% for men in Cohort II; and 1.1%, 17.6%, 6.3%, and 29.6% for women in Cohort II, respectively.

### 3.1. Validation Using Biomarkers as a Reference

**Figure 1:**
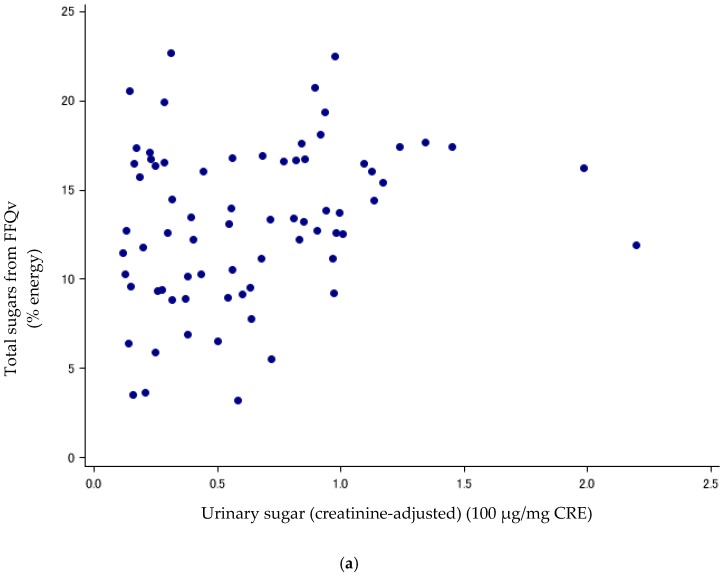

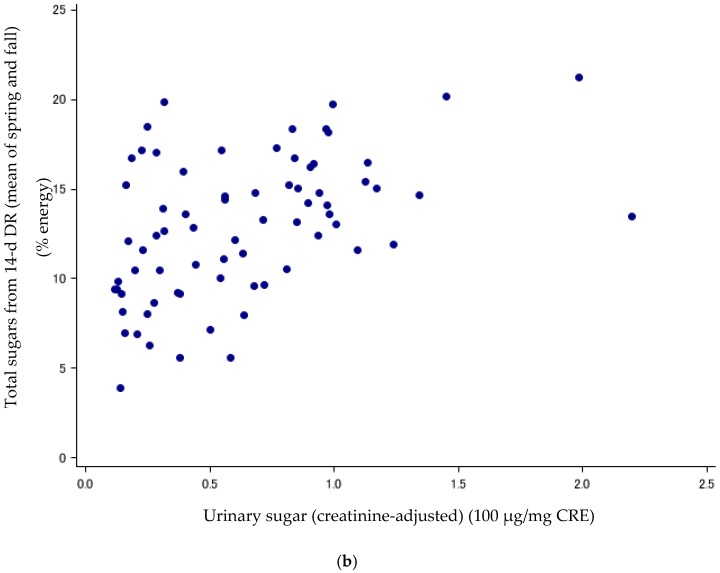
(**a**) Scatter plots between urinary sugars and total sugars from FFQv (*n* = 72, Cohort I). FFQv, food frequency questionnaire for validity. (**b**) Scatter plots between urinary sugars and total sugars from DR (*n* = 72, Cohort I). DR, dietary record.

Sugar intake assessed with the DR and FFQv is shown in Table 2. Urinary sugar concentrations were correlated with total sugars (% energy) from the FFQv (de-attenuated Spearman’s CC: r = 0.40, 95%CI: 0.19, 0.58) (Table 3; Figure 1a); and total sugars (% energy) from the 14-d DR (*r* = 0.89, 95%CI: 0.82, 0.93) (Table 3; Figure 1b). The σ^2^_ws_/σ^2^_bs_ ratios and ICCs, as measures of intra-subject variation, were high and low for urinary sugars, respectively (σ^2^_ws_/σ^2^_bs_ ratios: 5.62; ICCs: 0.15, *n* = 72).

For comparisons of the total sugars form the FFQv and urinary sugars based on the joint classification by quintile, 63% of the participants were classified into the same or adjacent categories, and 6.0% were classified into the extreme categories (Table S3-1). For details, 16 out of the participants (*n* = 72) were classified into the same, 45 were classified into the same or adjacent, while 4 were classified into the extreme categories (Tables S3-1 and S3-2).

### 3.2. Validation Using DR as a Reference

The major sources of total sugars were sucrose, glucose, and fructose. Total sugar intakes were higher among women than men. The SD of sugar intake from the FFQ tended to be larger than in the DR. Overall, total sugar intakes from the FFQv were over-estimated when compared to the DR (Table 2). For the energy-adjusted (% energy) total sugars, Spearman’s CCs (95%CI) were 0.64 (0.50, 0.74) for men and 0.48 (0.32, 0.61) for women in Cohort I; 0.62 (0.52, 0.71) for men and 0.37 (0.23, 0.49) for women in Cohort II (Table S4, Figure S1). Results became slightly weaker after adjusting for age, areas, occupations, BMI, total energy intake, smoking status, and alcohol intake; partial Spearman’s CCs (95%CI) were 0.57 (0.42, 0.69) for men and 0.41 (0.24, 0.55) for women in Cohort I; 0.56 (0.44, 0.65) for men and 0.34 (0.20, 0.47) for women in Cohort II (Table 4). The CCs were moderate, and higher in Cohort I than in Cohort II, and higher for men than for women. De-attenuated Spearman’s CCs based on the probit transformation method were slightly stronger (Table S5). Pearson CCs also showed moderate correlations, and de-attenuated Pearson CCs were slightly stronger (not shown in tables). Furthermore, in any of the cohorts by gender, the differences did not depend on the magnitude of the mean total sugar intakes (Figure S2).

For comparisons of the FFQv and DR sugars based on the cross classification by quintile, about 80% men and 70% women were classified into the same or adjacent categories of sugar intakes (total sugars), and less than 6.0% of men and women were classified into the opposite extreme categories (Table S6).

### 3.3. Reproducibility

For reproducibility, participants’ characteristics were described in previous studies [15,27]. For almost all of the sugars, estimated intakes from the FFQr were neither over- nor under-estimated when compared to the FFQv (Table 5). For total sugars (% energy), Spearman’s CCs (95%CI) were 0.63 (0.49, 0.73) for men and 0.55 (0.41, 0.67) for women in Cohort I; and 0.66 (0.55, 0.74) for men and 0.63 (0.52, 0.72) for women in Cohort II. The CCs were moderate and slightly lower for women (Table 6).

## 4. Discussion

We evaluated the validity and reproducibility of sugar intakes assessed by the FFQ in a subsample of the JPHC study. For validity, de-attenuated Spearman’s CC was 0.40 between total sugar intake from the FFQv and urinary sugar concentrations. Furthermore, after adjusting for age, areas, occupations, body mass index, total energy intake, smoking status and alcohol, partial correlations of sugar intakes between the FFQv and 28- or 14-d DR ranged from 0.34 to 0.57. These results suggested moderate validity of the FFQ. Compared with the 1-year interval FFQ, correlations ranged from 0.55 to 0.66, indicating moderate reproducibility. Our results for the JPHC study verified that it is possible to use the FFQ for the assessment of the health impacts of sugar intakes. It is expected that future studies will clarify the health impacts of sugar consumption in Japan.

The results of our study are in general agreement with previous studies. Smith et al. [28] assessed the validity of the FFQ by comparing it with three 4-d weighted DRs, and the reproducibility by comparing it with a 12–18-month interval FFQ. Spearman’s CCs of sugar intakes were 0.47 (energy-adjusted, 34 men and 45 women) for validity and 0.67 (96 men and 135 women) for reproducibility. Willett et al. [29] evaluated the validity and reproducibility of FFQ comparing it with 28-d DRs and 1-year interval FFQ, respectively, in 173 women. In the study, the Pearson CCs of sucrose intakes were 0.41 (energy-adjusted) and 0.71 for validity and reproducibility, respectively.

The SD of the total sugar intake assessed with the FFQv was almost double that of the DR, indicating that the between-person variation would be overestimated. Therefore, when we examine the association of total sugar intake from the FFQ with disease risks, such misclassification tends to attenuate relative risk estimates.

Except for glucose and galactose, women consumed larger amount of sugars than men according to the DR. The difference was remarkable in sucrose intake, because the contribution proportion of confectionaries (which were one of the main sources of sucrose) was higher in women. Furthermore, the % energy of free sugars in women was also higher than that for men. These characteristics in the source of sugars in women might affect the relationship between sugar intake and health conditions.

In the Bland-Altman plots, differences in total sugar intakes (% energy) between the FFQv and the DR did not differ based on the magnitude of the mean total sugar intakes (% energy). Moreover, FFQ estimates for total sugar intake were overestimated, especially in men.

Correlations between sugar intakes from the FFQv and DR among women in Cohort II were weaker than those in other groups. In previous studies, CCs between carbohydrate intakes estimated from the FFQv and DR among women in Cohort II were lower than for men (Cohort I, men: 0.56; women: 0.37; Cohort II, men: 0.59; women: 0.39) [15,23]. Because men tend to be unconcerned about their daily diets, it might have been easier for men to complete the FFQ, which requires simplified dietary habits [23].

Urinary sugars have been drawing attention as a useful biomarker not affected by measurement errors in self-reported measures [20] and the use of the same food composition table. We evaluated the validity of using urinary concentration of fructose and sucrose as an objective biomarker. In this study, sugar intakes from the FFQv were correlated with the mean concentrations of urinary sugars collected twice (spring and fall) (r = 0.40, 95%CI: 0.19, 0.58). The correlation between sugar intakes from the 7-day DR and urinary sugars, both collected in spring, was weak (r = 0.27, 95%CI: 0.04, 0.47), while the correlation between those collected in the fall was moderate (r = 0.46, 95%CI: 0.26, 0.62). For urinary sugars, σ^2^_ws_/σ^2^_bs_ was high and ICC was low; therefore, the concentrations of sugars in urine were likely to be influenced by within-subject variance and seasonal variations. Furthermore, a previous study [30] showed that participants who consumed higher added sugar resulted in better correlations between dietary sugar intakes and urinary sugar excretions (r = 0.77) than those who consumed lower added sugar (r = 0.15). Thus, the high consumption of sugars might have led to stronger correlations. In our study, total sugar intakes from the DR in the fall were higher than in spring due to increasing fruit intakes (interquartile range of total sugar intakes: 46.0–80.3 g/day in spring; 53.6–90.3 g/day in fall; fruit intakes: 65–192 g/day in spring; 120–273 g/day in fall; n = 72). Therefore, it can be speculated that the correlations between urinary sugars and the 7-d DR were higher in the fall than in spring, as a result of the seasonal variations in total sugar intakes. Of note is the correlation between sugar intakes and urinary sugars in a previous study [31] in which urine was collected daily based on a 30-day diet (r = 0.84, total sugars, n = 13). This seems to suggest that multiple measurements of urinary sugars lead to a high correlation between sugar intakes and urinary sugars and may be more useful than single or double measurements for examining the validity of sugar intakes.

Our study has several strengths. First, we complemented the standard tables of food composition for available carbohydrates with the substitution methods, because the tables do not cover all food items occurring in the FFQ and DR. Furthermore, we also included sugars added to foods during cooking. Accordingly, most of the food items and menus that contain non-negligible amount of sugars were included in the nutrient calculation. Secondly, we examined urinary sugars as an objective biomarker for validation. We found that urinary sugars were useful to some extent in evaluating the validity of the FFQ.

Despite these strengths, our study had some limitations. First, common errors in sugar intake assessments from the FFQ and the DR remained because we used the same food composition table for the nutritional calculation. In both the FFQ and the DR sugar intake estimation, we were unable to consider the heterogeneity of sugar contents in each food since our estimation was based only on the sugar content of foods on the standard tables of food composition. Therefore, the correlations between FFQ and DR might be overestimated. Furthermore, because both of the FFQ and DR are self-reported dietary assessments, the overestimations of the correlations also possibly existed. By contrast, urinary sugars were not affected by this limitation of the food composition tables [12] and the property of these dietary surveys. Our results showed a correlation between sugar intakes from the FFQ and urinary sugars, supporting the validity of the FFQ. Second, some foods in the FFQ and the DR were not assigned sugar contents and were not included in the calculations. However, we believe that this may not have seriously biased our estimates because we evaluated most food items that provide more than 1 g of carbohydrates per portion size. Third, because the dietary data in this study were collected before 2000, they may be different from contemporary dietary habits. Therefore, the results in this study might not be generalizable to studies conducted later. Fourth, correlations of sugar intakes and urinary sugar concentrations may differ by the form of the sugar in the food. Indeed, consistent with a previous report [32], urinary sugar concentrations were more strongly correlated with free sugar intakes measured by the DR than with other sugar intakes in our study (data not shown), suggesting that the validity of sugar intake by form may deserve further investigation.

## 5. Conclusions

We observed moderate correlations between sugar intakes from the FFQ and urinary sugar, and the DR, as well as between the two FFQs at yearly intervals. The FFQ used in the 5-year follow-up JPHC study may be useful in ranking individuals for sugar intakes in the JPHC study population. These findings suggest that the FFQ may be helpful in assessing the association of sugar intakes with health conditions in Japan.

## Figures and Tables

**Table 1:** Major food groups contributing to sugar intakes from the dietary records (Cohorts I and II).

Men (*n* = 276)		Women (*n* = 289)	
Food Groups	(%)	Food Groups	(%)
Total sugars			
Fruits	21.3	Fruits	24.4
Apples	(5.0)	Apples	(5.5)
Citrus	(3.9)	Citrus	(5.0)
Bananas	(3.0)	Japanese persimmons	(3.5)
Vegetables	14.3	Confectionaries	19.3
Onions	(2.1)	Traditional fresh and semi-dry confectionery	(10.8)
Carrots	(1.8)	Cake and pastry	(2.7)
Japanese Radishes	(1.8)	Traditional dry confectionery	(1.6)
Sugars and sweeteners	14.2	Milk and milk products	13.1
Sugars	(13.6)	Liquid milk	(8.6)
Honey and syrup	(0.6)	Yogurt	(2.4)
-	-	Ice cream	(1.5)
Confectionaries	13.4	Sugars and sweeteners	12.4
Traditional fresh & semi-dry confectionery	(8.1)	Sugars	(11.8)
Cake & pastry	(1.5)	Honey and syrup	(0.5)
Bun with filling	(1.3)	-	-
Milk and milk products	11.1	Vegetables	12.3
Liquid milk	(8.0)	Onions	(1.7)
Yogurt	(1.7)	Pumpkin and squash	(1.5)
Ice cream	(1.0)	Carrots	(1.5)
Non-alcoholic beverages	8.3	Non-alcoholic beverages	5.9
Carbonated beverage	(3.6)	Carbonated beverage	(1.8)
Coffee	(2.1)	Lactic acid bacteria beverage	(1.4)
Lactic acid bacteria beverage	(1.0)	Fruit drinks	(1.0)
Seasonings	4.8	Seasonings	3.7
Miso	(2.8)	Miso	(2.1)
Japanese Worcester sauce	(0.6)	-	-
Soy sauce	(0.5)	-	-
Alcohol	4.5		
Fermented alcoholic beverage	(2.5)		
Compound alcoholic beverage	(2.0)		
-	-		
Cereals	3.3		
Bread	(1.9)		
Rice	(0.8)		
Noodles	(0.6)		
Glucose			
Vegetables	26.9	Fruits	30.1
Fruits	20.8	Vegetables	28.0
Alcohol	19.4	Seasonings	14.3
Seasonings	15.1	Non-alcoholic beverages	8.4
Non-alcoholic beverages	8.7	Alcohol	8.4
Cereals	3.9	Cereals	4.1
Fructose			
Fruits	41.4	Fruits	48.2
Vegetables	32.8	Vegetables	28.3
Non-alcoholic beverages	13.1	Non-alcoholic beverages	11.1
Cereals	4.5	Cereals	4.6
Seasonings	3.6		
Galactose			
Seasonings	55.6	Milk and milk products	57.2
Milk and milk products	43.8	Seasonings	42.1
Sucrose			
Sugars and sweeteners	27.8	Confectionaries	34.7
Confectionaries	25.9	Sugars and sweeteners	22.6
Fruits	20.1	Fruits	21.3
Non-alcoholic beverages	7.9	Vegetables	4.9
Vegetables	5.9	Non-alcoholic beverages	4.5
Milk and milk products	3.0	Milk and milk products	4.1
Maltose			
Cereals	41.4	Cereals	32.5
Potatoes	21.5	Potatoes	27.0
Confectionaries	16.8	Confectionaries	22.3
Alcohol	8.8	Alcohol	6.3
Milk and milk products	4.2	Milk and milk products	5.4
Lactose			
Milk and milk products	93.0	Milk and milk products	93.0
Non-alcoholic beverages	3.2	Confectionaries	3.7
Starch			
Cereals	90.7	Cereals	85.0
Confectionaries	3.6	Confectionaries	7.0
		Potatoes	3.7

Food groups contributing to at least 3% of sugars intakes were listed. For total sugars, the top three contributing foods were listed. Non-alcoholic beverages category included 100% fruit juices (including reconstituted fruit juices), fruit drinks (less than 100% fruit juices), lactic acid bacteria beverages, coffee flavoured milk beverages, maccha, coffee, cocoa, and carbonated beverages.

**Table 2:** Sugar intakes assessed with DR for 28 or 14 days and FFQv in Cohort I & II and differences.

			DR			FFQv			Mean of Difference ^1^ (95%CI)	
			Mean	SD	Median	Mean	SD	Median		
Cohort I										
	Men (n = 102)									
		Total sugars ^2^ (g)	56.5	24.7	53.6	69.1	43.0	61.4	12.7	(5.3, 20.0)
		Glucose (g)	12.4	5.0	11.8	17.1	9.0	15.5	4.6	(3.2, 6.1)
		Fructose (g)	9.7	5.0	8.3	13.9	11.0	11.9	4.2	(2.4, 6.1)
		Galactose (g)	0.2	0.3	0.2	0.2	0.4	0.1	0.0	(−0.1, 0.0)
		Sucrose (g)	27.7	15.4	24.9	28.1	21.5	21.8	0.5	(−3.4, 4.3)
		Maltose (g)	0.9	0.5	0.8	1.1	0.6	0.9	0.1	(0.0, 0.3)
		Lactose (g)	5.6	4.6	4.7	8.8	10.2	7.3	3.2	(1.4, 5.0)
		Starch (g)	222.0	61.2	204.0	213.1	64.4	203.6	−8.9	(−18.7, 0.9)
		Energy (kcal)	2392	435	2372	2408	692	2357	16	(−105, 136)
		Protein (g)	91.1	15.6	90.1	88.1	35.6	82.2	−3.0	(−9.1, 3.1)
		Fat (g)	59.6	10.9	59.4	61.5	27.1	59.2	1.9	(−3.1, 7.0)
		Carbohydrate (g)	323.6	82.4	312.8	325.2	105.0	311.5	1.6	(−14.2, 17.3)
	Women (n = 113)									
		Total sugars ^2^ (g)	64.5	22.9	65.2	74.3	52.6	62.3	9.8	(0.7, 19.0)
		Glucose (g)	11.4	3.7	11.5	16.4	12.1	13.7	5.0	(2.9, 7.1)
		Fructose (g)	10.8	4.4	10.9	16.7	14.9	12.9	5.9	(3.2, 8.5)
		Galactose (g)	0.2	0.2	0.2	0.3	0.3	0.1	0.0	(0.0, 0.1)
		Sucrose (g)	34.1	14.7	33.5	30.4	24.7	23.4	−3.7	(−8.1, 0.8)
		Maltose (g)	1.1	0.6	1.0	1.2	0.8	0.9	0.0	(−0.1, 0.2)
		Lactose (g)	6.8	4.0	6.8	9.4	7.7	9.1	2.6	(1.5, 3.7)
		Starch (g)	161.6	37.4	160.7	180.1	51.5	172.8	18.5	(11.0, 26.0)
		Energy (kcal)	1856	321	1839	2054	846	1916	198	(54, 343)
		Protein (g)	75.5	13.0	75.9	82.0	44.1	72.7	6.5	(−1.2, 14.3)
		Fat (g)	53.1	10.4	52.5	59.4	32.4	51.7	6.3	(0.3, 12.3)
		Carbohydrate (g)	261.9	58.9	264.6	292.0	110.0	278.6	30.1	(12.5, 47.6)
Cohort II										
	Men (n = 174)									
		Total sugars ^2^ (g)	57.1	20.2	54.3	67.0	35.9	58.0	9.9	(5.1, 14.7)
		Glucose (g)	12.8	4.3	12.5	16.0	8.4	13.7	3.2	(2.1, 4.4)
		Fructose (g)	9.6	4.1	9.2	12.5	7.9	10.5	2.9	(1.9, 4.0)
		Galactose (g)	0.2	0.2	0.2	0.3	0.4	0.1	0.0	(0.0, 0.1)
		Sucrose (g)	27.3	12.9	24.5	27.4	18.5	24.6	0.1	(−2.3, 2.5)
		Maltose (g)	1.5	1.1	1.2	1.3	0.8	1.0	−0.2	(−0.4, −0.1)
		Lactose (g)	5.7	4.4	4.6	9.6	11.9	8.0	3.9	(2.3, 5.4)
		Starch (g)	200.6	45.2	192.8	197.0	60.6	184.2	−3.7	(−11.1, 3.8)
		Energy (kcal)	2268	358	2263	2291	690	2215	23	(−75, 121)
		Protein (g)	86.6	14.9	86.1	82.0	31.6	76.2	−4.7	(−9.5, 0.1)
		Fat (g)	54.8	11.7	54.3	59.6	26.1	54.3	4.9	(0.9, 8.9)
		Carbohydrate (g)	306.9	57.5	303.1	304.8	91.7	289.3	−2.1	(−14.2, 10.0)
	Women (*n* = 176)									
		Total sugars ^2^ (g)	63.9	19.7	62.4	69.0	39.4	60.8	5.1	(−0.2, 10.5)
		Glucose (g)	11.5	3.0	11.4	14.6	8.4	13.2	3.1	(1.9, 4.3)
		Fructose (g)	10.5	3.4	10.0	14.2	9.3	12.6	3.7	(2.4, 5.0)
		Galactose (g)	0.3	0.2	0.2	0.3	0.5	0.2	0.1	(0.0, 0.1)
		Sucrose (g)	32.3	13.6	30.9	28.3	19.3	24.6	−4.0	(−6.6, −1.4)
		Maltose (g)	1.8	1.3	1.4	1.2	0.8	1.1	−0.6	(−0.8, −0.4)
		Lactose (g)	7.5	4.4	6.9	10.3	10.9	9.3	2.8	(1.4, 4.2)
		Starch (g)	150.6	30.9	148.8	166.6	43.6	163.9	16.0	(10.1, 21.9)
		Energy (kcal)	1761	263	1753	1929	708	1794	167	(69, 266)
		Protein (g)	71.2	11.1	70.9	76.3	34.6	69.9	5.1	(0.2, 10.0)
		Fat (g)	48.1	9.9	47.9	57.6	30.2	51.0	9.5	(5.1, 13.9)
		Carbohydrate (g)	253.3	45.7	249.0	271.6	87.4	257.8	18.4	(6.6, 30.1)

DR, dietary record; FFQv, food frequency questionnaire for validity; SD, standard deviation; CI, confidence interval. ^1^ Mean of (intakes from FFQv—intakes from DR). ^2^ “Total sugars” represents the sum of the crude consumption of the following saccharides: glucose, fructose, galactose, sucrose, maltose, and lactose.

**Table 3:** The correlations between urinary sugars and dietary sugars by DR or FFQ (*n* = 72, Cohort I).

	Spearman’s Rank Correlation Coefficient					
	Crude ^1^		Adjusted ^2^		De-Attenuated ^3^	
	r	95%CI	r	95%CI	r	95%CI
FFQv vs. urine ^4^	0.25	(0.02, 0.45)	0.27	(0.04, 0.47)	0.40	(0.19, 0.58)
14-d DR ^5^ vs. urine ^4^	0.46	(0.25, 0.62)	0.48	(0.28, 0.64)	0.89	(0.82, 0.93)
7-d DR vs. urine (spring)	0.31	(0.08, 0.50)	0.24	(0.01, 0.44)	0.27	(0.04, 0.47)
7-d DR vs. urine (fall)	0.38	(0.16, 0.56)	0.41	(0.19, 0.58)	0.46	(0.26, 0.62)

DR, dietary record; FFQv, food frequency questionnaire for validity; r, correlation coefficient; CI, confidence interval. The σ2ws/σ2bs ratios: 5.62 and intra-class correlation (ICC): 0.15 for urinary sugars. ^1^ DR or FFQv (crude) vs. urinary sugars (crude). ^2^ DR or FFQv (energy-adjusted by nutritional density method (percentage of energy)) vs. urinary sugars (creatinine-adjusted). ^3^ Adjusted Spearman’s correlation coefficients were multiplied using probit transformation with regard to repeats of urinary sugar measures (twice) for FFQv vs. urine, repeats of both urinary sugar measures (twice) and DR measures (14 times) for 14-d DR vs. urine, and repeats of DR measures (7 times) for 7-d DR vs. urine. ^4^ Urinary sugar was calculated as the mean of the spring and fall values. ^5^ Dietary sugars from DR were calculated as the mean of the spring and fall values (the same seasons as when the urine was collected).

**Table 4:** Partial Correlations between FFQv and DR for 28 or 14 days (Validity).

			Partial Spearman’s Rank Correlation Coefficient ^1^					
			Crude		Residual Method ^2^		Density Method ^3^	
			r	95%CI	r	95%CI	r	95%CI
Cohort I								
	Men (*n* = 99)							
		Total sugars ^4^	0.49	(0.33, 0.63)	0.52	(0.36, 0.65)	0.57	(0.42, 0.69)
		Glucose	0.52	(0.36, 0.65)	0.56	(0.41, 0.68)	0.57	(0.42, 0.69)
		Fructose	0.54	(0.38, 0.67)	0.54	(0.39, 0.67)	0.55	(0.40, 0.68)
		Galactose	0.29	(0.10, 0.46)	0.48	(0.31, 0.62)	0.36	(0.17, 0.52)
		Sucrose	0.45	(0.28, 0.60)	0.46	(0.29, 0.60)	0.51	(0.35, 0.64)
		Maltose	0.41	(0.23, 0.56)	0.45	(0.27, 0.59)	0.46	(0.29, 0.60)
		Lactose	0.61	(0.48, 0.72)	0.54	(0.38, 0.66)	0.56	(0.41, 0.68)
		Starch	0.53	(0.38, 0.66)	0.42	(0.24, 0.57)	0.37	(0.19, 0.53)
		Protein	0.19	(−0.01, 0.37)	0.25	(0.06, 0.43)	0.25	(0.06, 0.43)
		Fat	0.28	(0.09, 0.46)	0.39	(0.21, 0.55)	0.40	(0.22, 0.55)
		Carbohydrate	0.37	(0.19, 0.53)	0.44	(0.26, 0.58)	0.46	(0.29, 0.60)
	Women (*n* = 113)							
		Total sugars ^4^	0.26	(0.07, 0.42)	0.36	(0.19, 0.51)	0.41	(0.24, 0.55)
		Glucose	0.19	(0.01, 0.37)	0.26	(0.08, 0.42)	0.29	(0.11, 0.45)
		Fructose	0.29	(0.11, 0.45)	0.31	(0.13, 0.47)	0.35	(0.17, 0.50)
		Galactose	0.51	(0.36, 0.63)	0.49	(0.33, 0.62)	0.51	(0.36, 0.63)
		Sucrose	0.23	(0.04, 0.39)	0.38	(0.21, 0.53)	0.35	(0.18, 0.50)
		Maltose	0.31	(0.13, 0.47)	0.41	(0.25, 0.55)	0.37	(0.20, 0.52)
		Lactose	0.69	(0.57, 0.77)	0.67	(0.56, 0.76)	0.66	(0.54, 0.75)
		Starch	0.30	(0.12, 0.46)	0.44	(0.28, 0.58)	0.32	(0.14, 0.48)
		Protein	0.18	(0.00, 0.36)	0.28	(0.10, 0.44)	0.27	(0.09, 0.43)
		Fat	0.20	(0.01, 0.37)	0.44	(0.28, 0.58)	0.42	(0.25, 0.56)
		Carbohydrate	0.20	(0.01, 0.37)	0.44	(0.28, 0.58)	0.39	(0.22, 0.54)
Cohort II								
	Men (*n* = 168)							
		Total sugars ^4^	0.42	(0.28, 0.53)	0.56	(0.45, 0.66)	0.56	(0.44, 0.65)
		Glucose	0.27	(0.13, 0.41)	0.44	(0.31, 0.56)	0.44	(0.30, 0.55)
		Fructose	0.42	(0.28, 0.53)	0.53	(0.41, 0.63)	0.52	(0.40, 0.62)
		Galactose	0.66	(0.57, 0.74)	0.64	(0.55, 0.73)	0.66	(0.56, 0.74)
		Sucrose	0.42	(0.29, 0.54)	0.52	(0.40, 0.62)	0.53	(0.41, 0.63)
		Maltose	0.17	(0.02, 0.31)	0.26	(0.11, 0.39)	0.29	(0.15, 0.42)
		Lactose	0.75	(0.68, 0.81)	0.74	(0.66, 0.80)	0.74	(0.66, 0.80)
		Starch	0.43	(0.30, 0.55)	0.55	(0.44, 0.65)	0.46	(0.33, 0.57)
		Protein	0.16	(0.01, 0.30)	0.41	(0.27, 0.53)	0.36	(0.23, 0.49)
		Fat	0.21	(0.06, 0.35)	0.52	(0.40, 0.62)	0.48	(0.35, 0.59)
		Carbohydrate	0.37	(0.23, 0.49)	0.55	(0.43, 0.65)	0.48	(0.36, 0.59)
	Women (*n* = 171)							
		Total sugars ^4^	0.23	(0.08, 0.37)	0.38	(0.25, 0.51)	0.34	(0.20, 0.47)
		Glucose	0.26	(0.12, 0.40)	0.30	(0.16, 0.43)	0.30	(0.16, 0.43)
		Fructose	0.31	(0.17, 0.44)	0.32	(0.18, 0.45)	0.32	(0.18, 0.45)
		Galactose	0.58	(0.47, 0.67)	0.62	(0.52, 0.70)	0.63	(0.53, 0.71)
		Sucrose	0.18	(0.03, 0.32)	0.33	(0.18, 0.45)	0.30	(0.16, 0.43)
		Maltose	0.17	(0.02, 0.31)	0.18	(0.04, 0.33)	0.19	(0.04, 0.33)
		Lactose	0.65	(0.55, 0.73)	0.72	(0.64, 0.78)	0.72	(0.64, 0.78)
		Starch	0.27	(0.13, 0.40)	0.39	(0.25, 0.51)	0.34	(0.20, 0.47)
		Protein	0.22	(0.07, 0.36)	0.30	(0.16, 0.43)	0.28	(0.13, 0.41)
		Fat	0.28	(0.14, 0.42)	0.41	(0.28, 0.53)	0.36	(0.22, 0.49)
		Carbohydrate	0.14	(−0.01, 0.28)	0.43	(0.30, 0.55)	0.39	(0.25, 0.51)

DR, dietary record; FFQv, food frequency questionnaire for validity; CC, correlation coefficient; CI, confidence interval. ^1^ Correlation coefficients were adjusted for age, area, occupation (primary industry, professionals and office workers, self-employed and others, unemployed), body mass index (BMI), total energy intake, smoking status (never, past, current), and alcohol (non-drinker, ≤ 4 days per week, ≥ 5 days per week). ^2^ Sugar and other nutrients intakes were adjusted for energy intake by residual model. ^3^ Sugar and other nutrients intakes were energy-adjusted using the density method (percentage of energy). ^4^ “Total sugars” represents the sum of the crude consumption of following saccharides: glucose, fructose, galactose, sucrose, maltose, and lactose.

**Table 5:** Sugar intakes assessed with FFQv and FFQr in Cohorts I and II and differences.

			FFQv			FFQr			Mean of Difference ^1^ (95%CI)	
			Mean	SD	Median	Mean	SD	Median		
Cohort I										
	Men (*n* = 101)									
		Total sugars ^2^ (g)	69.3	43.2	61.5	70.4	41.6	61.3	1.1	(−6.7, 9.0)
		Glucose (g)	17.1	9.0	15.5	17.9	11.1	15.7	0.8	(−1.1, 2.6)
		Fructose (g)	13.9	11.1	12.0	14.1	10.1	11.6	0.2	(−1.9, 2.2)
		Galactose (g)	0.2	0.4	0.1	0.2	0.2	0.1	0.0	(−0.1, 0.0)
		Sucrose (g)	28.2	21.6	21.9	27.9	20.3	23.5	−0.3	(−3.6, 3.0)
		Maltose (g)	1.1	0.6	0.9	1.2	1.4	0.9	0.2	(−0.1, 0.4)
		Lactose (g)	8.8	10.2	7.3	9.1	9.7	8.8	0.4	(−2.0, 2.8)
		Starch (g)	213.4	64.7	203.8	213.9	71.7	195.4	0.5	(−11.5, 12.6)
		Energy (kcal)	2403	694	2354	2418	741	2415	14	(−129, 158)
		Protein (g)	87.7	35.5	81.5	87.9	35.0	84.6	0.2	(−6.2, 6.6)
		Fat (g)	61.5	27.2	59.1	62.2	29.1	57.1	0.7	(−4.9, 6.3)
		Carbohydrate (g)	325.4	105.5	316.3	326.8	107.8	304.3	1.4	(−17.8, 20.6)
	Women (*n* = 108)									
		Total sugars ^2^ (g)	74.2	52.9	62.8	74.9	32.6	67.9	0.7	(−8.2, 9.6)
		Glucose (g)	16.4	12.2	13.7	16.5	7.7	14.4	0.1	(−1.8, 2.1)
		Fructose (g)	16.7	15.0	13.1	16.2	8.8	13.8	−0.4	(−2.9, 2.0)
		Galactose (g)	0.2	0.2	0.1	0.3	0.3	0.2	0.0	(0.0, 0.1)
		Sucrose (g)	30.7	25.1	23.6	31.1	16.3	29.0	0.5	(−3.8, 4.8)
		Maltose (g)	1.2	0.8	0.9	1.2	0.7	1.0	0.0	(−0.1, 0.2)
		Lactose (g)	9.1	7.1	9.1	9.6	10.1	8.0	0.5	(−1.3, 2.3)
		Starch (g)	179.9	52.4	172.6	182.8	46.8	177.4	2.9	(−4.7, 10.5)
		Energy (kcal)	2048	860	1914	2082	601	1959	33	(−109, 176)
		Protein (g)	81.5	44.6	71.9	81.9	29.4	76.8	0.4	(−7.4, 8.1)
		Fat (g)	59.3	32.9	51.6	61.5	27.2	54.2	2.3	(−3.8, 8.3)
		Carbohydrate (g)	291.4	111.8	277.5	295.4	78.9	279.1	4.0	(−13.5, 21.5)
Cohort II										
	Men (*n* = 143)									
		Total sugars ^2^ (g)	64.8	31.3	55.9	68.1	34.2	62.1	3.4	(−1.1, 7.8)
		Glucose (g)	16.0	8.5	13.7	16.5	9.0	14.9	0.5	(−0.5, 1.6)
		Fructose (g)	12.4	7.7	10.4	13.0	8.6	10.3	0.5	(−0.5, 1.6)
		Galactose (g)	0.2	0.3	0.1	0.2	0.3	0.1	0.0	(0.0, 0.0)
		Sucrose (g)	25.9	14.7	24.1	27.6	16.4	25.3	1.7	(−0.3, 3.7)
		Maltose (g)	1.3	0.8	1.0	1.3	0.8	1.2	0.1	(0.0, 0.2)
		Lactose (g)	8.9	9.2	8.4	9.5	9.4	8.6	0.5	(−1.3, 2.4)
		Starch (g)	191.6	57.4	182.5	202.2	58.9	193.9	10.6	(3.4, 17.8)
		Energy (kcal)	2251	650	2167	2387	740	2210	135	(44, 227)
		Protein (g)	80.4	30.1	74.9	86.6	33.7	81.2	6.2	(1.9, 10.5)
		Fat (g)	59.0	25.2	54.0	64.8	28.9	58.9	5.8	(1.6, 10.0)
		Carbohydrate (g)	296.4	85.2	279.6	311.9	90.4	296.5	15.6	(4.9, 26.2)
	Women (*n* = 146)									
		Total sugars ^2^ (g)	68.6	36.6	59.1	72.2	42.1	63.2	3.6	(−2.1, 9.4)
		Glucose (g)	14.7	8.2	13.2	15.6	9.8	13.8	0.9	(−0.4, 2.1)
		Fructose (g)	14.2	9.1	12.5	15.1	12.4	12.8	0.9	(−0.8, 2.6)
		Galactose (g)	0.4	0.5	0.2	0.3	0.4	0.2	0.0	(−0.1, 0.1)
		Sucrose (g)	27.4	16.9	23.9	29.0	19.0	24.6	1.6	(−1.0, 4.1)
		Maltose (g)	1.2	0.7	1.1	1.3	0.9	1.1	0.1	(−0.1, 0.2)
		Lactose (g)	10.8	11.6	9.4	11.0	9.6	9.4	0.2	(−1.7, 2.1)
		Starch (g)	163.0	38.7	163.5	170.3	40.1	169.5	7.3	(0.9, 13.7)
		Energy (kcal)	1911	621	1769	2036	635	1893	125	(36, 214)
		Protein (g)	75.5	30.1	69.8	80.8	30.4	75.3	5.3	(1.3, 9.4)
		Fat (g)	57.5	27.2	50.8	63.1	27.0	58.0	5.5	(1.4, 9.7)
		Carbohydrate (g)	267.9	78.6	254.8	280.4	82.8	266.2	12.4	(1.0, 23.8)

FFQv, food frequency questionnaire for validity; FFQr, food frequency questionnaire for reproducibility; SD, standard deviation; CI, confidence interval. ^1^ Mean of (intakes from FFQr - intakes from FFQv). ^2^ “Total sugars” represents the sum of the crude consumption of the following saccharides: glucose, fructose, galactose, sucrose, maltose, and lactose.

**Table 6:** Correlations between FFQv and FFQr (Reproducibility).

			Spearman’s Rank Correlation Coefficient						
			Crude		Energy-Adjusted (Residual) ^1^		Energy-Adjusted (Density) ^2^		ICC for FFQ
			r	95%CI	r	95%CI	r	95%CI	
Cohort I									
	Men (*n* = 101)								
		Total sugars ^3^	0.61	(0.48, 0.72)	0.53	(0.38, 0.66)	0.63	(0.49, 0.73)	0.66
		Glucose	0.65	(0.52, 0.75)	0.55	(0.40, 0.68)	0.64	(0.51, 0.74)	0.63
		Fructose	0.65	(0.52, 0.75)	0.56	(0.41, 0.68)	0.60	(0.46, 0.71)	0.61
		Galactose	0.73	(0.62, 0.81)	0.42	(0.24, 0.57)	0.66	(0.53, 0.76)	0.67
		Sucrose	0.75	(0.65, 0.82)	0.70	(0.59, 0.79)	0.76	(0.67, 0.83)	0.75
		Maltose	0.58	(0.43, 0.69)	0.68	(0.56, 0.77)	0.68	(0.56, 0.78)	0.50
		Lactose	0.63	(0.50, 0.74)	0.51	(0.35, 0.64)	0.53	(0.37, 0.65)	0.50
		Starch	0.69	(0.57, 0.78)	0.62	(0.49, 0.73)	0.51	(0.35, 0.64)	0.46
		Energy	0.47	(0.30, 0.61)					
		Protein	0.56	(0.40, 0.68)	0.47	(0.30, 0.61)	0.56	(0.41, 0.68)	0.60
		Fat	0.51	(0.35, 0.64)	0.60	(0.46, 0.72)	0.59	(0.44, 0.70)	0.59
		Carbohydrate	0.66	(0.53, 0.76)	0.65	(0.52, 0.75)	0.60	(0.46, 0.71)	0.54
	Women (*n* = 108)								
		Total sugars ^3^	0.65	(0.52, 0.75)	0.57	(0.43, 0.69)	0.55	(0.41, 0.67)	0.62
		Glucose	0.69	(0.57, 0.78)	0.55	(0.41, 0.67)	0.61	(0.48, 0.72)	0.63
		Fructose	0.65	(0.53, 0.75)	0.55	(0.40, 0.67)	0.60	(0.47, 0.71)	0.63
		Galactose	0.59	(0.45, 0.70)	0.51	(0.36, 0.64)	0.52	(0.37, 0.64)	0.54
		Sucrose	0.67	(0.55, 0.76)	0.61	(0.48, 0.72)	0.61	(0.48, 0.72)	0.59
		Maltose	0.69	(0.57, 0.77)	0.64	(0.51, 0.74)	0.67	(0.54, 0.76)	0.68
		Lactose	0.74	(0.64, 0.82)	0.74	(0.64, 0.82)	0.75	(0.65, 0.82)	0.71
		Starch	0.71	(0.60, 0.79)	0.56	(0.42, 0.68)	0.60	(0.46, 0.71)	0.56
		Energy	0.69	(0.58, 0.78)					
		Protein	0.68	(0.56, 0.77)	0.42	(0.25, 0.56)	0.49	(0.33, 0.62)	0.47
		Fat	0.67	(0.55, 0.76)	0.58	(0.44, 0.69)	0.59	(0.46, 0.70)	0.59
		Carbohydrate	0.72	(0.62, 0.80)	0.55	(0.40, 0.67)	0.58	(0.44, 0.69)	0.57
Cohort II									
	Men (*n* = 143)								
		Total sugars ^3^	0.63	(0.52, 0.72)	0.65	(0.55, 0.74)	0.66	(0.55, 0.74)	0.64
		Glucose	0.71	(0.62, 0.78)	0.63	(0.52, 0.72)	0.66	(0.55, 0.74)	0.63
		Fructose	0.64	(0.53, 0.73)	0.61	(0.50, 0.71)	0.62	(0.51, 0.71)	0.63
		Galactose	0.72	(0.63, 0.79)	0.68	(0.57, 0.76)	0.69	(0.60, 0.77)	0.76
		Sucrose	0.68	(0.58, 0.76)	0.67	(0.57, 0.75)	0.68	(0.57, 0.76)	0.67
		Maltose	0.63	(0.51, 0.72)	0.67	(0.57, 0.75)	0.68	(0.58, 0.76)	0.78
		Lactose	0.68	(0.58, 0.76)	0.69	(0.60, 0.77)	0.70	(0.60, 0.77)	0.64
		Starch	0.69	(0.59, 0.77)	0.64	(0.54, 0.73)	0.62	(0.51, 0.71)	0.64
		Energy	0.59	(0.47, 0.69)					
		Protein	0.60	(0.49, 0.70)	0.61	(0.50, 0.70)	0.60	(0.49, 0.70)	0.68
		Fat	0.56	(0.44, 0.66)	0.63	(0.52, 0.72)	0.61	(0.50, 0.70)	0.64
		Carbohydrate	0.64	(0.53, 0.73)	0.69	(0.59, 0.77)	0.66	(0.56, 0.75)	0.70
	Women (*n* = 146)								
		Total sugars ^3^	0.64	(0.53, 0.73)	0.59	(0.47, 0.68)	0.63	(0.52, 0.72)	0.48
		Glucose	0.60	(0.49, 0.70)	0.41	(0.26, 0.54)	0.45	(0.32, 0.57)	0.45
		Fructose	0.58	(0.46, 0.67)	0.36	(0.21, 0.50)	0.45	(0.31, 0.57)	0.45
		Galactose	0.65	(0.55, 0.74)	0.61	(0.50, 0.71)	0.65	(0.55, 0.74)	0.55
		Sucrose	0.63	(0.52, 0.72)	0.53	(0.41, 0.64)	0.57	(0.45, 0.67)	0.55
		Maltose	0.69	(0.60, 0.77)	0.71	(0.62, 0.78)	0.69	(0.59, 0.76)	0.65
		Lactose	0.76	(0.68, 0.82)	0.79	(0.72, 0.84)	0.79	(0.72, 0.85)	0.73
		Starch	0.53	(0.40, 0.64)	0.51	(0.38, 0.62)	0.58	(0.46, 0.68)	0.56
		Energy	0.60	(0.48, 0.69)					
		Protein	0.65	(0.54, 0.73)	0.52	(0.39, 0.63)	0.59	(0.47, 0.69)	0.54
		Fat	0.60	(0.48, 0.69)	0.51	(0.38, 0.62)	0.50	(0.36, 0.61)	0.37
		Carbohydrate	0.58	(0.46, 0.68)	0.50	(0.37, 0.61)	0.50	(0.37, 0.62)	0.48

DR, dietary record; FFQv, food frequency questionnaire for validity; r, correlation coefficient; CI, confidence interval; ICC, intra-class correlation coefficient. ^1^ Sugar and other nutrients intakes were adjusted for energy intake by residual model. ^2^ Sugar and other nutrients intakes were energy-adjusted using the density method (percentage of energy). ^3^ “Total sugars” represents the sum of the crude consumption of the following saccharides: glucose, fructose, galactose, sucrose, maltose, and lactose.

## Supplementary Materials

The following are available online at https://www.mdpi.com/2072-6643/11/3/554/s1, Table S1: Substituted food items in the FFQ, Table S2: Major foods contributing to sugar intake according to the DR (Cohorts I & II), Table S3-1. Comparison of FFQ for sugar intakes with urinary sugars based on cross classification by quintile (%), Table S3-2. Frequency and means of FFQ for sugar intakes with urinary sugars based on cross classification by quintile (Cohort I, *n* = 72), Table S4: Correlations between FFQv and DR for 28 or 14 days, Table S5: Rank correlation coefficients between % energy of sugar intake assessed using the DR for 28 days and FFQv in Cohorts I and II using the probit transformation method with correction for measurement error, Table S6: Comparison of FFQv with DR for sugar intakes based on cross classification by quintile (%), Figure S1: Scatter plots between total sugars from FFQ and DR, Figure S2: Bland-Altman plot for the comparison of FFQ and DR in measuring the total sugar intake.
