# Supplementary material for: Validity and Reproducibility of a Self-Administered Food Frequency Questionnaire for the Assessment of Sugar Intake in Middle-Aged Japanese Adults
Source: Nutrients. 2019 Mar 5;11(3):554. doi: 10.3390/nu11030554 (PMC6470835; doi:10.3390/nu11030554)
Supplement: Supplementary file 1 [file nutrients-11-00554-s001.pdf]

Table S1. Substituted food items in the FFQ

Methods	Number of food items	Foods
Using different parts of the same species, similar species, or same species with different cooking or purification methods	18	Vitamin-enriched rice, akamiso (a type of miso), pickled radish, pickled green vegetable, pickled plum, pickled Chinese cabbage, pickled cucumber, pickled eggplants, leaf mustard, Swiss chard, sponge gourd, mugwort, hassaku orange(a type of citrus fruits), citrus iyo (a type of citrus fruits), dried wakame (seaweed), margarine, mayonnaise, and sauce
Prepared by dietitians using the recipes	6	Grilled eel, chikuwa, kamaboko, salad dressing, canned coffee, powder corn soup

FFQ, frequency food questionnaire

Table S2. Major foods contributing to sugar intake according to the DR (Cohorts I and II)

	Men (n=276)			Women (n=289)		
**Sugars**	**Food groups**	**Major contributing foods**	**Proportion (%)**	**Food groups**	**Major contributing foods**	**Proportion (%)**
Total sugars						
	Fruits		21.3	Fruits		24.4
		Apples	(5.0)		Apples	(5.5)
		Citrus	(3.9)		Citrus	(5.0)
		Bananas	(3.0)		Japanese persimmons	(3.5)
		Japanese persimmons	(2.8)		Bananas	(2.8)
		Watermelon	(1.2)		Watermelon	(1.4)
		Grapes	(0.9)		Grapes	(1.0)
		Muskmelons	(0.9)		Muskmelons	(0.9)
		Pears	(0.7)		Pears	(0.7)
		Jam	(0.6)		Jam	(0.7)
		Peaches	(0.5)		Peaches	(0.7)
	Vegetables		14.3	Confectionaries		19.3
		Onions	(2.1)		Traditional fresh and semi-dry confectionery	(10.8)
		Carrots	(1.8)		Cake and pastry	(2.7)
		Japanese radishes	(1.8)		Traditional dry confectionery	(1.6)
		Pumpkin and squash	(1.5)		Bun with filling	(1.5)
		Tomatoes	(1.4)		Pudding and chilled dessert	(1.0)
		Cabbage	(1.2)		Chocolate	(0.8)
		Cucumber	(0.7)		Biscuits	(0.7)
		Eggplant	(0.7)			
		Welsh onions	(0.5)	Milk and milk products		13.1
		Chinese cabbage	(0.5)		Liquid milk	(8.6)
		Sweet corn	(0.5)		Yogurt	(2.4)
					Ice cream	(1.5)
	Sugars and sweeteners		14.2			
		Sugars	(13.6)	Sugars and sweeteners		12.4
		Honey and syrup	(0.6)		Sugars	(11.8)
					Honey and syrup	(0.5)
	Confectionaries		13.4			
		Traditional fresh & semi-dry confectionery	(8.1)	Vegetables		12.3
		Cake & pastry	(1.5)		Onions	(1.7)

	Bun with filling	(1.3)		Pumpkin and squash	(1.5)
	Traditional dry confectionery	(1.1)		Carrots	(1.5)
	Chocolate	(0.5)		Japanese radishes	(1.4)
				Tomatoes	(1.4)
Milk and milk products		11.1		Cabbage	(0.9)
	Liquid milk	(8.0)		Cucumber	(0.6)
	Yogurt	(1.7)		Sweet corn	(0.5)
	Ice cream	(1.0)		Eggplant	(0.5)
Non-alcoholic beverages		8.3	Non-alcoholic beverages		5.9
	Carbonated beverage	(3.6)		Carbonated beverage	(1.8)
	Coffee	(2.1)		Lactic acid bacteria beverage	(1.4)
	Lactic acid bacteria beverage	(1.0)		Fruit drinks	(1.0)
	Fruit drinks	(1.0)		Coffee	(0.9)
	100 percent fruit juice	(0.6)		100 percent fruit juice	(0.8)
Seasonings		4.8	Seasonings		3.7
	Miso	(2.8)		Miso	(2.1)
	Japanese Worcester sauce	(0.6)			
	Soy sauce	(0.5)			
Alcohol		4.5			
	Fermented alcoholic beverage	(2.5)			
	Compound alcoholic beverage	(2.0)			
Cereals		3.3			
	Bread	(1.9)			
	Rice	(0.8)			
	Noodles	(0.6)			
Glucose					
Vegetables		26.9	Fruits		30.1
	Japanese radishes	(4.2)		Japanese persimmons	(7.3)
	Onions	(4.1)		Citrus	(5.9)
	Cabbage	(3.0)		Apples	(3.5)
	Tomatoes	(2.9)		Grapes	(2.8)
	Carrots	(2.4)		Bananas	(2.6)
	Pumpkin and squash	(1.5)		Watermelon	(2.0)
	Cucumber	(1.5)		Pears	(0.7)

	Eggplant	(1.5)		Muskmelons	(0.7)
	Chinese cabbage	(1.3)		Strawberries	(0.6)
	Welsh onions	(1.0)		Pineapple	(0.6)
	Sweet corn	(0.5)			
			Vegetables		28.0
Fruits		20.8		Onions	(4.1)
	Japanese persimmons	(4.6)		Japanese radishes	(4.1)
	Citrus	(3.6)		Tomatoes	(3.6)
	Apples	(2.6)		Cabbage	(2.9)
	Bananas	(2.3)		Carrots	(2.4)
	Grapes	(2.0)		Pumpkin and squash	(2.0)
	Watermelon	(1.4)		Cucumber	(1.6)
	Pears	(0.6)		Eggplant	(1.4)
	Muskmelons	(0.5)		Chinese cabbage	(1.2)
	Pineapple	(0.5)		Welsh onions	(1.0)
				Sweet corn	(0.7)
Alcohol		19.4			
	Fermented alcoholic beverage	(11.1)	Seasonings		14.3
	Compound alcoholic beverage	(8.3)		Miso	(10.5)
				Soy sauce	(1.5)
Seasonings		15.1		Japanese Worcester sauce	(1.2)
	Miso	(11.1)		Tomato products	(0.8)
	Soy sauce	(1.6)			
	Japanese Worcester sauce	(1.4)	Non-alcoholic beverages		8.4
	Tomato products	(0.8)		Carbonated beverage	(3.3)
				Lactic acid bacteria beverage	(1.9)
Non-alcoholic beverages		8.7		Fruit drinks	(1.7)
	Carbonated beverage	(5.3)		100 percent fruit juice	(1.3)
	Fruit drinks	(1.3)			
	Lactic acid bacteria beverage	(1.2)	Alcohol		8.4
	100 percent fruit juice	(0.9)		Compound alcoholic beverage	(7.8)
				Fermented alcoholic beverage	(0.5)
Cereals		3.9			
	Bread	(2.3)	Cereals		4.1
	Rice	(1.5)		Bread	(2.9)
				Rice	(1.1)
Fructose					
Fruits		41.4	Fruits		48.2

Apples	(14.5)	Apples	(16.3)
Japanese persimmons	(5.7)	Japanese persimmons	(7.2)
Citrus	(5.3)	Citrus	(7.1)
Watermelon	(3.9)	Watermelon	(4.6)
Bananas	(2.8)	Grapes	(2.9)
Grapes	(2.6)	Bananas	(2.6)
Pears	(2.0)	Pears	(2.1)
Muskmelons	(0.7)	Strawberries	(0.8)
Strawberries	(0.6)	Muskmelons	(0.8)
Pineapple	(0.6)	Pineapple	(0.6)
Vegetables	32.8	Vegetables	28.3
Onions	(5.2)	Tomatoes	(4.4)
Tomatoes	(4.3)	Onions	(4.2)
Japanese radishes	(4.3)	Japanese radishes	(3.4)
Cabbage	(3.0)	Carrots	(2.5)
Carrots	(3.0)	Cabbage	(2.4)
Cucumber	(2.2)	Pumpkin and squash	(2.0)
Pumpkin and squash	(1.8)	Cucumber	(1.9)
Eggplant	(1.8)	Eggplant	(1.4)
Chinese cabbage	(1.3)	Chinese cabbage	(1.0)
Welsh onions	(1.3)	Welsh onions	(1.0)
Sweet corn	(0.6)	Sweet corn	(0.7)
Non-alcoholic beverages	13.1	Non-alcoholic beverages	11.1
Carbonated beverage	(7.6)	Carbonated beverage	(4.2)
Fruit drinks	(2.2)	Fruit drinks	(2.5)
100 percent fruit juice	(1.8)	100 percent fruit juice	(2.3)
Lactic acid bacteria beverage	(1.6)	Lactic acid bacteria beverage	(2.1)
Cereals	4.5	Cereals	4.6
Bread	(4.4)	Bread	(4.5)
Seasonings	3.6		
Japanese Worcester sauce	(1.5)		
Miso	(1.1)		
Tomato products	(0.9)		
Galactose			

	Seasonings		55.6	Milk and milk products		57.2
		Soy sauce	(34.9)		Yogurt	(54.7)
		Miso	(20.2)		Ice cream	(1.7)
					Cheeses	(0.5)
	Milk and milk products		43.8			
		Yogurt	(41.7)	Seasonings		42.1
		Ice cream	(1.2)		Soy sauce	(26.5)
		Cheeses	(0.8)		Miso	(15.3)
Sucrose						
	Sugars and sweeteners		27.8	Confectionaries		34.7
		Sugars	(27.8)		Traditional fresh and semi-dry confectionery	(20.0)
					Cake and pastry	(4.9)
	Confectionaries		25.9		Traditional dry confectionery	(2.9)
		Traditional fresh and semi-dry confectionery	(15.9)		Bun with filling	(2.6)
		Cake and pastry	(2.9)		Pudding and chilled dessert	(1.5)
		Bun with filling	(2.4)		Biscuits	(1.3)
		Traditional dry confectionery	(2.2)		Chocolate	(1.2)
		Chocolate	(0.9)			
		Biscuits	(0.8)	Sugars and sweeteners		22.6
		Pudding and chilled dessert	(0.6)		Sugars	(22.6)
	Fruits		20.1	Fruits		21.3
		Citrus	(4.5)		Citrus	(5.4)
		Bananas	(4.2)		Apples	(4.2)
		Apples	(4.0)		Bananas	(3.6)
		Japanese persimmons	(1.6)		Japanese persimmons	(2.0)
		Muskmelons	(1.3)		Muskmelons	(1.3)
		Jam	(1.0)		Jam	(1.1)
		Peaches	(0.8)		Peaches	(1.0)
		Watermelon	(0.5)		Watermelon	(0.5)
		Pears	(0.5)		Pears	(0.5)
		Pineapple	(0.5)		Pineapple	(0.5)
	Non-alcoholic beverages		7.9	Vegetables		4.9
		Coffee	(3.9)		Pumpkin and squash	(1.7)
		Carbonated beverage	(2.3)		Carrots	(1.2)
		Lactic acid bacteria beverage	(0.8)		Sweet corn	(0.5)
		Fruit drinks	(0.6)		Onions	(0.5)

	Vegetables		5.9	Non-alcoholic beverages		4.5
		Pumpkin and squash	(1.7)		Coffee	(1.6)
		Carrots	(1.6)		Carbonated beverage	(1.0)
		Onions	(0.6)		Lactic acid bacteria beverage	(1.0)
		Sweet corn	(0.6)		Fruit drinks	(0.5)
	Milk and milk products		3.0	Milk and milk products		4.1
		Yogurt	(1.4)		Yogurt	(2.1)
		Ice cream	(1.3)		Ice cream	(1.8)
Maltose						
	Cereals		41.4	Cereals		32.5
		Noodles	(20.8)		Bread	(18.6)
		Bread	(20.0)		Noodles	(13.5)
		Wheat flour	(0.5)			
				Potatoes		27.0
	Potatoes		21.5		Starch sweeteners	(0.6)
		Sweet potato	(21.5)			
				Confectionaries		22.3
	Confectionaries		16.8		Traditional fresh and semi-dry confectionery	(14.1)
		Traditional fresh and semi-dry confectionery	(11.1)		Candy	(3.3)
		Traditional dry confectionery	(2.2)		Traditional dry confectionery	(3.2)
		Candy	(1.6)		Bun with filling	(1.4)
		Bun with filling	(1.4)			
				Alcohol		6.3
	Alcohol		8.8		Compound alcoholic beverage	(6.3)
		Compound alcoholic beverage	(8.8)			
				Milk and milk products		5.4
	Milk and milk products		4.2		Ice cream	(4.8)
		Ice cream	(3.6)		Sherbet	(0.5)
		Sherbet	(0.6)			
Lactose						
	Milk and milk products		93.0	Milk and milk products		93.0
		Liquid milk	(80.0)		Liquid milk	(76.9)
		Yogurt	(7.5)		Yogurt	(9.0)
		Ice cream	(2.4)		Ice cream	(3.1)
		Milk powder	(2.1)		Milk powder	(3.1)

	Coffee whitener, cream	(0.6)		Coffee whitener, cream	(0.5)
Non-alcoholic beverages		3.2	Confectionaries		3.7
	Coffee	(2.0)		Chocolate	(1.4)
	Lactic acid bacteria beverage	(1.2)		Bun with filling	(0.8)
				Cake and pastry	(0.8)
				Pudding and chilled dessert	(0.5)
Starch					
Cereals		90.7	Cereals		85.0
	Rice	(75.5)		Rice	(67.6)
	Noodles	(9.0)		Noodles	(8.9)
	Bread	(3.7)		Bread	(5.7)
	Wheat	(2.0)		Wheat	(2.4)
Confectionaries		3.6	Confectionaries		7.0
	Traditional fresh and semi-dry confectionery	(1.3)		Traditional fresh and semi-dry confectionery	(2.6)
	Traditional dry confectionery	(0.9)		Traditional dry confectionery	(1.5)
	Bun with filling	(0.6)		Cake and pastry	(1.2)
	Cake and pastry	(0.5)		Bun with filling	(1.0)
				Biscuits	(0.6)
			Potatoes		3.7
				Potato	(1.9)
				Sweet potato	(1.2)

DR, dietary record
Foods that contributed to at least 0.5% have been listed.

Table S3-1. Comparison of FFQ for sugar intakes with urinary sugars based on cross classification by quintile (%)

	Cohort I n=72 (Men: n=27, Women: n=45)		
	**Same category[1]**	**Same or Adjacent category[2]**	**Extreme category[3]**
Total sugars[4,5]	22 (n=16)	63 (n=45)	6 (n=4)

[1] Percentage of participants whose sugar intakes from FFQ and urinary sugars were classified into the same category by quintile.

[2] Percentage of participants whose sugar intakes from FFQ and urinary sugars were classified into the same or adjacent category by quintile.

[3] Percentage of participants whose sugar intakes from FFQ and urinary sugars were classified into the extreme (lowest or highest) category by quintile.

[4] "Total sugars" was the sum of the crude consumption of the following saccharides: glucose, fructose, galactose, sucrose, maltose, and lactose.

[5] Sugar intakes were adjusted using the density method and urinary sugars were adjusted by urinary creatinine concentration.

FFQ, food frequency questionnaire.

Table S3-2. Frequency and means of FFQ for sugar intakes with urinary sugars based on cross classification by quintile (Cohort I, n=72)

		Sugar intakes from FFQv (% energy)[1]				
	Means (SD)	6.7 (2.2)	10.7 (1.0)	13.3 (0.6)	16.2 (0.6)	19.0 (2.0)
**Urinary sugar (creatinine-adjusted)** [1]	0.17 (0.04)	3	4	1	3	3
	0.32 (0.05)	4	4	2	3	2
	0.57 (0.08)	5	4	2	2	1
	0.85 (0.08)	1	2	5	3	4
	1.26 (0.38)	1	1	4	4	4

FFQv, food frequency questionnaire for validity; SD, standard deviation

Dark grey cells represent the number of participants whose sugar intakes from FFQv and urinary sugars were classified into the same category by quintile.; Light grey cells represent the number of participants whose sugar intakes from FFQv and urinary sugars were classified into the adjacent category by quintile.; Shaded cells represent the number of participants whose sugar intakes from FFQv and urinary sugars were classified into the extreme (lowest or highest) category by quintile.

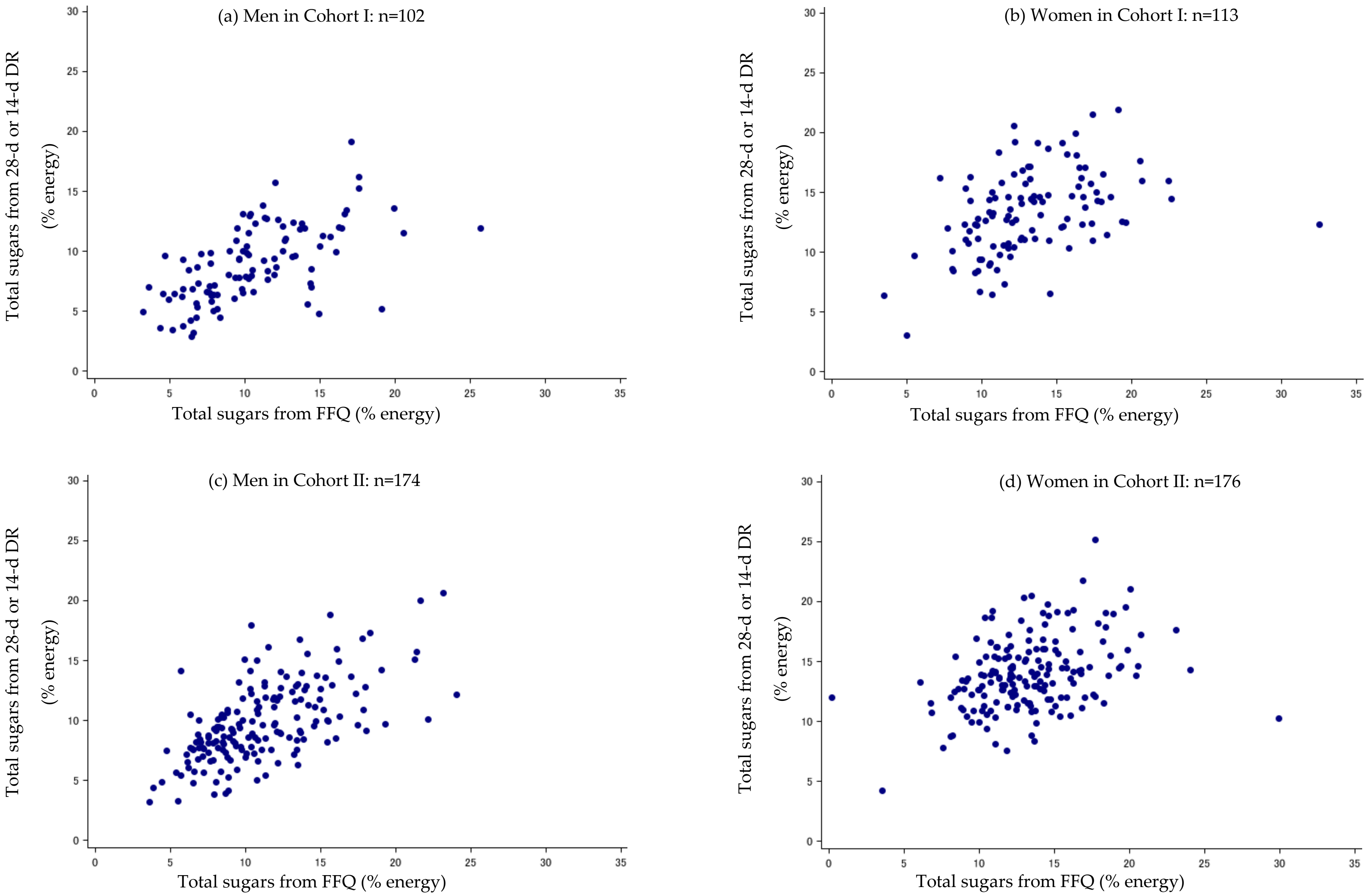

Figure S1. Scatter plots between total sugars from FFQ and DR

The regression coefficients: β=0.58 (p<0.001), men in Cohort I; β=0.49 (p<0.001), women in Cohort I; β=0.61 (p<0.001), men in Cohort II; β=0.26 (p<0.001), women in Cohort II. The percent energy of total sugar intake from the FFQ and DR were log-transformed for the regression analysis.

Table S4. Correlations between FFQv and DR for 28- or 14-days

	Spearman's rank Correlation Coefficient						
	Crude		Energy-adjusted (residual)[1]		Energy-adjusted (density)[2]		ICC
	r	95% CI	r	95% CI	r	95% CI	for DR
Cohort I							
Men (n=102)							
Total sugars[3]	0.64	(0.51, 0.74)	0.57	(0.43, 0.69)	0.64	(0.50, 0.74)	0.43
Glucose	0.67	(0.54, 0.76)	0.57	(0.43, 0.69)	0.63	(0.49, 0.73)	0.27
Fructose	0.59	(0.44, 0.70)	0.56	(0.42, 0.68)	0.58	(0.43, 0.69)	0.27
Galactose	0.55	(0.40, 0.67)	0.53	(0.38, 0.66)	0.56	(0.41, 0.68)	0.44
Sucrose	0.61	(0.48, 0.72)	0.55	(0.40, 0.67)	0.61	(0.47, 0.72)	0.41
Maltose	0.47	(0.31, 0.61)	0.45	(0.28, 0.60)	0.46	(0.29, 0.60)	0.13
Lactose	0.66	(0.54, 0.76)	0.60	(0.45, 0.71)	0.61	(0.47, 0.72)	0.43
Starch	0.74	(0.64, 0.82)	0.52	(0.36, 0.65)	0.47	(0.30, 0.61)	0.37
Energy	0.53	(0.37, 0.65)					
Protein	0.46	(0.29, 0.60)	0.29	(0.10, 0.46)	0.22	(0.02, 0.39)	0.21
Fat	0.32	(0.14, 0.49)	0.53	(0.37, 0.66)	0.49	(0.32, 0.62)	0.25
Carbohydrate	0.71	(0.59, 0.79)	0.60	(0.46, 0.71)	0.62	(0.48, 0.72)	0.42
Women (n=113)							
Total sugars[3]	0.52	(0.37, 0.64)	0.31	(0.13, 0.46)	0.48	(0.32, 0.61)	0.36
Glucose	0.48	(0.32, 0.61)	0.32	(0.15, 0.48)	0.42	(0.25, 0.56)	0.18
Fructose	0.51	(0.36, 0.63)	0.36	(0.18, 0.51)	0.46	(0.30, 0.59)	0.22
Galactose	0.67	(0.55, 0.76)	0.60	(0.47, 0.71)	0.64	(0.52, 0.74)	0.23
Sucrose	0.48	(0.32, 0.61)	0.33	(0.16, 0.49)	0.43	(0.27, 0.57)	0.32
Maltose	0.42	(0.26, 0.56)	0.43	(0.26, 0.57)	0.41	(0.24, 0.55)	0.13
Lactose	0.69	(0.58, 0.78)	0.66	(0.54, 0.75)	0.65	(0.52, 0.74)	0.34
Starch	0.58	(0.44, 0.69)	0.45	(0.29, 0.59)	0.34	(0.17, 0.49)	0.29
Energy	0.41	(0.25, 0.56)					

Protein	0.38	(0.21, 0.53)	0.25	(0.07, 0.42)	0.21	(0.03, 0.38)	0.17
Fat	0.20	(0.02, 0.37)	0.46	(0.30, 0.59)	0.45	(0.29, 0.59)	0.22
Carbohydrate	0.58	(0.45, 0.69)	0.43	(0.27, 0.57)	0.41	(0.24, 0.55)	0.29
Cohort II							
Men (n=174)							
Total sugars[3]	0.45	(0.32, 0.56)	0.62	(0.52, 0.70)	0.62	(0.52, 0.71)	0.42
Glucose	0.40	(0.27, 0.52)	0.53	(0.41, 0.63)	0.52	(0.40, 0.62)	0.30
Fructose	0.45	(0.32, 0.56)	0.57	(0.46, 0.66)	0.57	(0.46, 0.66)	0.33
Galactose	0.59	(0.49, 0.68)	0.60	(0.50, 0.69)	0.61	(0.51, 0.70)	0.45
Sucrose	0.41	(0.28, 0.53)	0.54	(0.42, 0.63)	0.54	(0.43, 0.64)	0.35
Maltose	0.30	(0.16, 0.43)	0.40	(0.27, 0.52)	0.44	(0.31, 0.55)	0.20
Lactose	0.76	(0.69, 0.82)	0.75	(0.68, 0.81)	0.77	(0.70, 0.82)	0.48
Starch	0.54	(0.43, 0.64)	0.64	(0.55, 0.72)	0.58	(0.47, 0.67)	0.39
Energy	0.37	(0.24, 0.49)					
Protein	0.29	(0.15, 0.42)	0.35	(0.22, 0.48)	0.34	(0.20, 0.47)	0.24
Fat	0.25	(0.10, 0.38)	0.54	(0.43, 0.64)	0.49	(0.37, 0.60)	0.30
Carbohydrate	0.46	(0.34, 0.57)	0.69	(0.60, 0.76)	0.67	(0.58, 0.74)	0.42
Women (n=176)							
Total sugars[3]	0.25	(0.11, 0.39)	0.39	(0.26, 0.51)	0.37	(0.23, 0.49)	0.30
Glucose	0.29	(0.14, 0.42)	0.34	(0.21, 0.47)	0.36	(0.22, 0.48)	0.20
Fructose	0.28	(0.14, 0.41)	0.30	(0.16, 0.43)	0.31	(0.17, 0.44)	0.20
Galactose	0.59	(0.48, 0.68)	0.63	(0.54, 0.71)	0.64	(0.55, 0.72)	0.28
Sucrose	0.22	(0.08, 0.36)	0.32	(0.18, 0.45)	0.30	(0.16, 0.43)	0.24
Maltose	0.24	(0.10, 0.38)	0.24	(0.10, 0.38)	0.26	(0.11, 0.39)	0.15
Lactose	0.67	(0.58, 0.74)	0.71	(0.62, 0.77)	0.71	(0.63, 0.78)	0.40
Starch	0.44	(0.31, 0.55)	0.45	(0.32, 0.56)	0.37	(0.23, 0.49)	0.38
Energy	0.26	(0.11, 0.39)					
Protein	0.33	(0.20, 0.46)	0.31	(0.17, 0.44)	0.26	(0.11, 0.39)	0.21
Fat	0.32	(0.18, 0.45)	0.48	(0.36, 0.59)	0.37	(0.23, 0.49)	0.26

Carbohydrate	0.31	(0.17, 0.44)	0.47	(0.35, 0.58)	0.37	(0.24, 0.49)	0.29

DR, dietary record; FFQv, food frequency questionnaire for validity; r, correlation coefficient; CI, confidence interval; ICC, intra-class correlation coefficient

[1] Sugar and other nutrients intakes were adjusted for energy intake by residual model.

[2] Sugar and other nutrients intakes were energy-adjusted using the density method (percentage of energy).

[3] "Total sugars" was the sum of the crude consumption of the following saccharides: glucose, fructose, galactose, sucrose, maltose, and lactose.

Table S5. Rank correlation coefficients between %energy of sugar intake assessed using the DR for 28 days and FFQv in Cohorts I and II using the probit transformation method with correction for measurement error

	Cohort I Men (n=75), Women (n=85)				Cohort II Men (n=174), Women (n=176)			
	Observed Spearman's rank CC		Corrected Spearman's rank CC		Observed Spearman's rank CC		Corrected Spearman's rank CC	
	r	(95% CI)	r	(95% CI)	r	(95% CI)	r	(95% CI)
Men								
Total sugars [1, 2]	0.58	(0.40, 0.71)	0.65	(0.50, 0.77)	0.60	(0.49, 0.69)	0.66	(0.56, 0.73)
Glucose [2]	0.59	(0.41, 0.72)	0.73	(0.61, 0.82)	0.48	(0.36, 0.59)	0.56	(0.45, 0.65)
Fructose [2]	0.54	(0.36, 0.68)	0.72	(0.59, 0.81)	0.55	(0.43, 0.64)	0.61	(0.51, 0.69)
Galactose [2]	0.47	(0.27, 0.63)	0.77	(0.66, 0.85)	0.52	(0.40, 0.62)	0.64	(0.55, 0.72)
Sucrose [2]	0.58	(0.41, 0.71)	0.65	(0.49, 0.76)	0.56	(0.44, 0.65)	0.63	(0.53, 0.71)
Maltose [2]	0.51	(0.32, 0.66)	0.77	(0.66, 0.85)	0.55	(0.43, 0.64)	0.68	(0.59, 0.75)
Lactose [2]	0.69	(0.55, 0.80)	0.81	(0.71, 0.88)	0.69	(0.60, 0.76)	0.76	(0.69, 0.82)
Starch	0.45	(0.25, 0.62)	0.51	(0.32, 0.66)	0.58	(0.47, 0.67)	0.64	(0.55, 0.72)
Women								
Total sugars [1, 2]	0.39	(0.19, 0.56)	0.46	(0.27, 0.61)	0.38	(0.24, 0.50)	0.44	(0.31, 0.55)
Glucose [2]	0.24	(0.03, 0.43)	0.30	(0.09, 0.48)	0.40	(0.26, 0.51)	0.47	(0.35, 0.58)
Fructose [2]	0.27	(0.06, 0.45)	0.34	(0.14, 0.52)	0.38	(0.24, 0.50)	0.44	(0.31, 0.55)
Galactose [2]	0.41	(0.21, 0.57)	0.55	(0.38, 0.68)	0.50	(0.38, 0.60)	0.63	(0.54, 0.71)
Sucrose [2]	0.37	(0.17, 0.54)	0.43	(0.24, 0.59)	0.31	(0.17, 0.44)	0.37	(0.24, 0.49)
Maltose [2]	0.45	(0.26, 0.60)	0.59	(0.43, 0.71)	0.43	(0.30, 0.54)	0.53	(0.42, 0.63)
Lactose [2]	0.69	(0.56, 0.79)	0.86	(0.79, 0.91)	0.66	(0.57, 0.74)	0.74	(0.67, 0.80)
Starch	0.41	(0.22, 0.57)	0.51	(0.34, 0.66)	0.36	(0.22, 0.48)	0.41	(0.28, 0.52)

DR, dietary record; FFQv, food frequency questionnaire for validity; CC, correlation coefficient; CI, confidence interval

Participants living in Ishikawa area in Cohort I were excluded from the analysis because they completed 14 days of DR.

[1] "Total sugars" was the sum of the crude consumption of the following saccharides: glucose, fructose, galactose, sucrose, maltose, and lactose.

[2] Sugars intakes were adjusted by using the density method (percentage of energy).

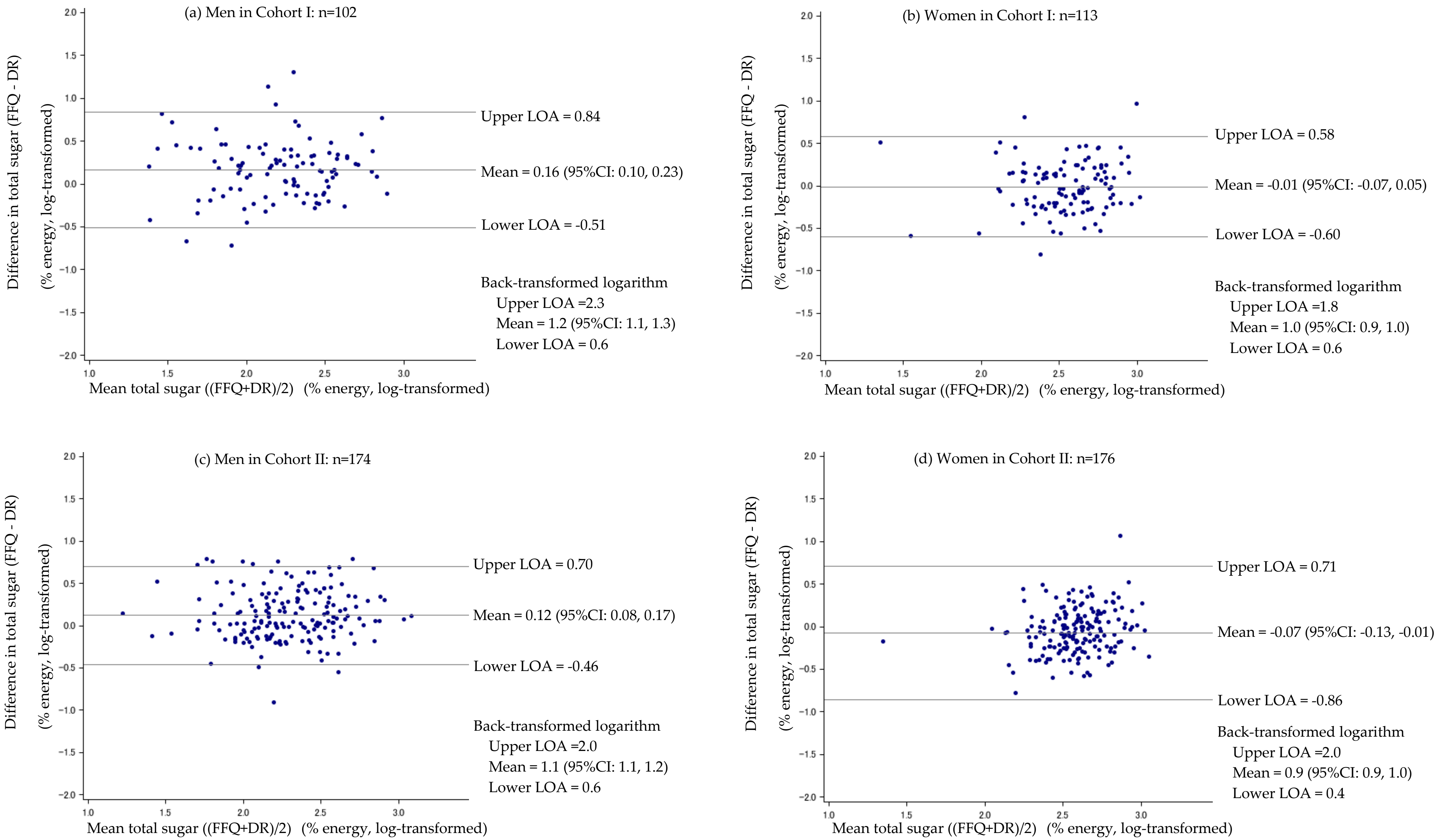

Figure S2. Bland-Altman plot for the comparison of the FFQ and DR in measuring the total sugar intake
FFQ, food frequency questionnaire; DR, dietary record; LOA, limit of agreement

Table S6. Comparison of FFQv with DR for sugar intakes based on cross classification by quintile (%)

	Cohort I Men (n=102), Women (n=113)			Cohort Ⅱ Men (n=174), Women (n=176)		
	Same category[1]	Same or Adjacent categories[2]	Extreme category[3]	Same category[1]	Same or Adjacent categories[2]	Extreme category[3]
Men						
Total sugars[4,5]	36	81	3	34	75	1
Glucose[5]	31	75	1	34	71	1
Fructose[5]	30	77	0	36	73	1
Galactose[5]	34	71	1	40	80	1
Sucrose[5]	28	78	2	32	75	2
Maltose[5]	32	72	1	32	69	3
Lactose[5]	35	82	1	45	87	1
Starch	35	74	3	37	72	1
Women						
Total sugars[4,5]	22	69	2	28	66	3
Glucose[5]	27	70	2	30	65	3
Fructose[5]	33	68	2	28	60	3
Galactose[5]	36	80	0	35	78	0
Sucrose[5]	29	72	3	33	64	3
Maltose[5]	20	68	4	26	60	6
Lactose[5]	43	78	1	41	87	1
Starch	35	64	4	27	67	3

FFQv, food frequency questionnaire for validity; DR, dietary record

[1] Percentage of participants whose sugar intakes from FFQ and DR were classified into the same category by quintile.

[2] Percentage of participants whose sugar intakes from FFQ and DR were classified into the same or adjacent category by quintile.

[3] Percentage of participants whose sugar intakes from FFQ and DR were classified into the extreme (lowest or highest) category by quintile.

[4] "Total sugars" was the sum of the crude consumption of the following saccharides: glucose, fructose, galactose, sucrose, maltose, and lactose.

[5] Sugars intakes were adjusted for using the density method.
